# Draft Genome Sequence of *Streptomyces* sp. Strain PBH53, Isolated from an Urban Environment

**DOI:** 10.1128/genomeA.00859-15

**Published:** 2015-07-30

**Authors:** Jessica T. Gosse, Patrick Hill, Scot E. Dowd, Christopher N. Boddy

**Affiliations:** a Department of Chemistry and Biomolecular Sciences, University of Ottawa, Ottawa, Canada; b Department of Biology, University of Ottawa, Ottawa, Canada; c MR DNA, Molecular Research LP, Shallowater, Texas, USA

## Abstract

We report the draft genome sequence of *Streptomyces* sp. strain PBH53, a strain isolated from an urban transit station in Ottawa, Canada. The analysis of the genome using the bioinformatics tool antiSMASH showed the presence of many unique natural product biosynthetic pathways.

## GENOME ANNOUNCEMENT

Sediments from urban environments, such as streets, contain diverse microbial communities (1) enriched in actinomycetes (2) and possessing unique secondary metabolite biosynthetic pathways (3).Urban sites may thus be a useful source for discovery of novel secondary metabolites. As the *Streptomyces* genus is a prolific producer of secondary metabolites (4), we sequenced the genome of a representative species isolated from an urban environment.

Sediment was taken from a bus shelter floor at the University of Ottawa, Ottawa, Canada, in the summer of 2010. Sediment was diluted in sterile water, plated on Difco actinomycete isolation agar (5), and incubated at 50°C for 72 h. Colonies with actinomycetal appearance were isolated and purified. The 16S rRNA gene was amplified and sequenced from the isolate PBH53, confirming that it belongs to the *Streptomyces* genus. PCR amplification using type I polyketide synthase (PKS)-specific primers confirmed that the strain encoded one or more polyketide biosynthetic pathways (6).

Genomic DNA was obtained from cultivation of PBH53 in yeast malt-extract medium (7). Genomic DNA was isolated using a Wizard genomic DNA purification kit (Promega). Sequencing on an Illumina Miseq DNA sequencer was performed at MR DNA (Shallowater, TX). The library for each sample was prepared using a Nextera DNA sample preparation kit (Illumina) following the manufacturer’s instructions. The library (12.5 pM) was sequenced using a 600-cycle v3 reagent kit (Illumina), with an average sequencing coverage of 50×. An initial annotation was made using the Rapid Annotation using Subsystems Technology (RAST) server (8). The draft genome contains 9,153,597 nucleotides with G+C content of 71.83%. A total of 167 contigs were obtained, with 102 containing protein-encoding genes. Seventy-six RNA loci were identified. Annotation using Glimmer (9) provided 15,123 putative genes.

The sequence was examined using antiSMASH 3.0.0 (10), and 47 secondary metabolite biosynthetic gene clusters were identified. Most are predicted to be involved in polyketide or nonribosomal peptide biosynthesis, including 9 type I PKS clusters, 2 type II PKS clusters, and 2 type III PKS cluster as well as 8 nonribosomal peptide synthetase (NRPS) gene clusters and 10 mixed PKS-NRPS or PKSI-PKSII clusters. A number of the predicted PKS and NRPS appeared to be gene cluster fragments. The remaining clusters were predicted to encode biosynthetic pathways for 2 terpenes, 3 siderophores, a lantipeptide, 3 bacteriocins, ectoin, and 2 melanins. Four clusters were identified but had no predicted compound family. Two predicted pathways showed high homology to known biosynthetic gene clusters. A siderophore gene cluster showed 80% similarity to the desferrioxamine B gene cluster, and the putative ectoin gene cluster showed 75% similarity to a known ectoin biosynthetic cluster.

The *Streptomyces* sp. PBH53 genome sequence provides information on a prolific secondary metabolite producer isolated from an urban environment. The large number of unique secondary metabolite biosynthetic gene clusters suggests that urban environments may be a promising site for new natural product discovery.

### Nucleotide sequence accession number.

This whole-genome shotgun project has been deposited at DDBJ/EMBL/GenBank under the accession number CP011799. The version described in this paper is the first version.
